# Current Trends in Multidisciplinary Approaches to Understanding Consumer Preference and Acceptance of Food Products

**DOI:** 10.3390/foods9101380

**Published:** 2020-09-29

**Authors:** Derek Victor Byrne

**Affiliations:** Food Quality Perception and Society Science Team, iSENSE Lab, Department of Food Science, Faculty of Technical Sciences, Aarhus University, 8200 Aarhus, Denmark; derekv.byrne@food.au.dk

**Keywords:** food preference, consumer, sensory perception, food choice, multidisciplinary

## Abstract

Acceptance and preference of the sensory properties of foods are among the most important criteria determining food choice. Sensory perception and our response to food products and finally food choice itself are affected by a myriad of intrinsic as well as extrinsic factors. The pressing question is, how do these factors specifically affect our acceptance and preference for foods, both in and of themselves, and in combination in various contexts, both fundamental and applied? In addition, which factors overall play the largest role in how we perceive and behave towards food in daily life? Finally, how can these factors be utilized to affect our preferences and final acceptance of real food and food products from industrial production and beyond for healthier eating? A closer look at trends in research showcasing the influence that these factors and our senses have on our perception and affective response to food products and our food choices is timely. Thus, in this Special Issue collection “Consumer Preferences and Acceptance of Food Products”, we bring together articles which encompass the wide scope of multidisciplinary research in the space related to the determination of key factors involved linked to fundamental interactions, cross-modal effects in different contexts and eating scenarios, as well as studies that utilize unique study design approaches and methodologies.

## 1. Introduction

The application of the human senses in studying consumer preferences and acceptance of food products has become increasingly multi- and cross-disciplinary in recent years. Moreover, sensory and consumer science is now more widely applicable than ever to a multitude of food and eating scenarios, including both intrinsic (to the food itself) and extrinsic (non-food cues) factors that influence food choice and eating behavior [1]. 

Acceptance and preference of the sensory properties of foods have been and are still among the most important criteria determining food choice [2,3,4,5,6]. There is much empirical research showcasing the effect that our senses have on our perception, affective response to food products and our food choices [7,8,9]. This effect of the senses is of course also affected by both the aforementioned intrinsic food product factors as well as extrinsic factors in a multitude of manners, both independently and in synergy [1]. 

The pressing question is how do these factors specifically affect our acceptance and preference for foods, both in and of themselves and in combination in various contexts, both fundamental and applied. In addition, there is the question of which of these factors overall play the largest role in how we perceive and behave towards food in daily life. Finally, there is the question of how intrinsic and extrinsic factors can be utilized to affect our preferences and final acceptance of real food and food products from industrial production and beyond for healthier eating. A closer look at trends in research showcasing the influence that external and internal influences and our senses have on our perception and affective response to food products and our food choices is therefore timely.

Thus, in this Special Issue collection “Consumer Preferences and Acceptance of Food Products” we bring together articles which encompass the wide scope of multidisciplinary research and perspectives in the space related to the determination of key factors involved. The articles included can be considered to cover stakeholders in the perception chain, from ‘the Senses’ regarding fundamental interactions [10,11,12], on to ‘Physiological responses’ [13,14], ‘Food choice’ itself, [15,16] and on to studies looking at ‘Purchasing decision processes’ [17,18], and finally to key factors in relation to behaviors in the ‘Market itself’ [19]. Moreover, we include an in-depth review of extrinsic vs. intrinsic factors themselves in a sweetness in beverage context which brings a unique perspective to beverage design for the future [1].

## 2. A Synopsis of Special Issues Research

### 2.1. The Senses

Thus, regarding ‘the Senses’, Bertelsen et al. (2020) examined the area of individual differences in sweetness ratings and cross-modal aroma-taste interactions. The authors indicated that aroma–taste interactions, which are believed to occur due to previous co-exposure (concurrent presence of aroma and taste), are suggested as a strategy to aid sugar reduction in food and beverages. However, co-exposures might be influenced by individual differences. The authors therefore hypothesized that aroma–taste interactions vary across individuals [10]. Moreover, Bertelsen et al. (2020) investigated how individual differences (gender, age, and sweet liker status) influenced the effect of aroma on sweetness intensity among young adults. Consumers were clustered according to their sweet liker status based on their liking for the samples [10]. Although sweet taste ratings were found to vary with the sweet liker status, aroma enhanced the sweetness ratings similarly across clusters. As a result, Bertelsen et al. (2020) suggested that these results call for more targeted product development in order to aid sugar reduction.

In addition, in relation to ‘the Senses’, Klotz et al. (2020) looked at the influence of the brewing temperature on the taste of espresso coffee. The context presented by the authors was that very hot (>65 °C) beverages such as espresso have been evaluated by the International Agency for Research on Cancer (IARC) as ‘likely’ carcinogenic to humans. For this reason, research into lowering beverage temperature without compromising its quality or taste is important. In two sensory trials using a triangle test methodology, brewing temperatures of 80 °C vs. 128 °C and 80 °C vs. 93 °C were compared. Most tasters were clearly unable to distinguish between 80 and 93 °C. The authors proposed that the results indicate that the possibility of decreasing the potential health hazards of very hot beverages exists by simply lower brewing temperatures to levels where tasters do not detect a difference [11].

In another included publication looking at the senses, Włodarska et al. (2020) specifically studied the visual system and factors influencing consumers’ perceptions of food, in this case with apple juice using sensory and visual attention methods. At its core, the authors’ aim was to evaluate the influence of intrinsic product characteristics and extrinsic packaging-related factors on the food quality perception [12]. The results show that brand and package information have a large impact on consumers’ sensory perceptions and generate high sensory expectations. An innovative visual attention tracking technique was used in online experiments to identify packages and label areas on individual packages, which attracted consumer attention. During an online shelf test, consumers mostly focused on not from concentrate juices from local producers, which were perceived as more natural, healthy, and expensive than juices reconstituted from concentrate. When individual labels were analyzed, consumers predominantly focused on nutritional data, brand name, and information about the type of product [12]. Włodarska et al. (2020) concluded that the present results confirm a large impact of information and visual stimuli related to packaging on product perception.

### 2.2. Physiological Responses

Relating to ‘Physiological responses’ and the senses, Szczygiel et al. (2020) looked at the effect of sleep curtailment on hedonic responses to liquid and solid food. The authors’ premise was that it is currently unclear whether changes in sweet taste perception of model systems after sleep curtailment extended to complex food matrices. Therefore, the primary objective of this study was to use a novel solid oat-based food (crisps) and an oat-based beverage stimulus sweetened with sucralose to assess changes in taste perception after sleep curtailment using a single-channel electroencephalograph [13]. Szczygiel et al. (2020) contended that overall, sweeter versions of the oat products were liked more after sleep curtailment. While the effect of sleep curtailment on sweet liking did not differ between sweet liking classification categories, sleep curtailment resulted in decreased texture liking in the solid oat crisps for sweet non-likers but not in the oat beverage. The authors concluded that these findings illustrate the varied effects of sleep on hedonic response in complex food matrices and possible mechanisms by which insufficient sleep can lead to sensory-moderated increases in energy intake [13].

In addition, Duerlund et al. (2020) uniquely looked at dynamic changes in post-ingestive sensations after the consumption of a breakfast meal high in protein or carbohydrate. The authors presented how post-ingestive sensations can provide a more comprehensive picture of the eating experience than mere satiety measurements. This study aimed to quantify the dynamics of different post-ingestive sensations after food intake and study the effect of protein and carbohydrate on hedonic and post-ingestive responses [14]. Subjects were served a breakfast meal high in protein (HighPRO) or high in carbohydrate (HighCHO). The results show a significant main effect of time for all post-ingestive sensations. HighCHO induced higher hedonic responses compared to HighPRO, as well as higher ratings for post-ingestive sensations such as satisfaction, food joy, overall wellbeing and fullness. HighPRO, on the other hand, induced higher ratings for sweet desire post intake. Duerlund et al. (2020) overall proposed that the development of sensations after a meal might be important for consumers’ following food choices and for extra calorie intake.

### 2.3. Food Choice

On to Food choice itself, Ohlhausen and Langen (2020) investigated how a combination of nudges decreases sustainable food choices out-of-home, utilizing food decoy effects (DE) and descriptive name labels (DNL). The authors reported the results from three consecutive studies focusing on the comparison of the effectiveness of different nudges and their combinations to increase sustainable food choices out of the home. The nudges compared are the use of descriptive name labels for the most sustainable dish of a choice set (menu) and the decoy effect, created by adding a less attractive decoy dish to a more attractive target dish with the goal of increasing the choice frequency of the target dish. The authors concluded that a combination of DNLs and the DE is not recommended for fostering sustainable food choices. Pure DNLs were more efficient in increasing the choice frequency of the more sustainable meal, whereas the decoy effect resulted in decreased choice frequencies. Also of note, regional and sustainable DNLs were favored by consumers [15].

Also in relation to food choice, Yeh et al. (2020) looked at the role of trust in explaining food choice where the authors combine a discrete choice experiment (DCE) and attribute best–worst scaling (BWS). The analysis was based on a sample of 459 Taiwanese consumers and focuses on red sweet peppers. The results of the DCE latent class analysis for the product attributes show that four segments may be distinguished [16]. Yeh et al. (2020) concluded that linking the DCE with the attitudinal dimensions reveals that consumers’ attitude and trust significantly explain class membership and, therefore, consumers’ preferences for different credence attributes.

### 2.4. Purchasing Behavior

Within the purchasing intention area, Park et al. (2020) investigated factors influencing purchasing of low-sodium and low-sugar products. The authors’ basis for this study was linked to the fact that sodium and sugar intake in South Korea exceeds recommended levels and as a result the government and food industry have been attempting to reduce the amount of sodium and sugar in food products, as in many other countries. For this study, two online survey-based experiments were conducted: one using soy sauce to represent a sodium-based product and the other using yogurt to represent a sugar-based product [17]. The significant variables that influenced the purchase intention for both were the consumers’ previous low-sodium/low-sugar product choices and their propensity for food neophobia. Moreover, the lower the consumer′s unhealthy = tasty intuition (UTI), the higher the purchase intention for the low-sodium soy sauce, but UTI did not act as a significant variable for the low-sugar yogurt. Park et al. (2020) concluded that the results demonstrate that government interventions for low-sodium products and low-sugar products should be differentiated to have impact.

Moreover, regarding purchasing decisions, Massaglia et al. (2020) looked at consumer preference heterogeneity evaluation in fruit and vegetable purchasing decisions. This study assesses consumer preferences during fruit and vegetable (FV) sales, considering the sociodemographic variables of individuals together with their choice of point of purchase. A choice experiment was conducted in two metropolitan areas in Northwest Italy. The relative importance assigned by consumers to 12 fruit and vegetable product attributes, including both intrinsic and extrinsic quality cues, was assessed by using the best–worst scaling (BWS) methodology [18]. The BWS results show that “origin”, “seasonality”, and “freshness” were the most preferred attributes that Italian consumers took into account for purchases, while no importance was given to “organic certification”, “variety”, or “brand”. Massaglia et al. (2020) concluded that their research demonstrates that age, average annual income, and families with children are all discriminating factors that influence consumer preference and behavior, in addition to affecting which point of purchase where the consumer prefers to acquire FV products.

### 2.5. Market Factors

Melovic et al. (2020) provided an overview and an analysis of market factors influencing consumers’ preferences and acceptance of organic food products, presenting key recommendations for the optimization of what is a developing market in Italy. Considering the benefits of the organic production system, it is recognized as one of the main drivers of future economic development [19]. However, the imbalance between demand and supply at the local market level represents one of the serious obstacles that prevents its future growth. Therefore, this article examined the key factors related to the main elements of the offer that have the strongest impact on consumer preferences and acceptance of organic food products. Furthermore, this article provided insight into some of the sensory properties of the offer that are important to consumers [19]. Finally, Melovic et al. (2020) gave recommendations for the optimization of the offerings on the organic food market based on the analysis of the influence of each of the elements (product, price, distribution, and promotion) on consumer acceptance of organic products and making purchasing decisions.

Finally, this Special Issue collection includes a comprehensive review by Wang et al. (2020), bringing together a comprehensive body of research on the role of intrinsic and extrinsic sensory factors, focused on sweetness perception of food and beverages. The authors showed that when it comes to eating and drinking per se, multiple factors from diverse sensory modalities have been shown to influence multisensory flavor perception and liking [1]. These factors have previously been strictly divided into either those that are intrinsic to the food itself (e.g., food colour, aroma, texture), or those that are extrinsic to it (e.g., related to the packaging, receptacle or external environment). Wang et al. (2020) demonstrated that given the obvious public health need for specifically sugar reduction, their review aimed to compare the relative influences of product-intrinsic and product-extrinsic factors on the perception of sweetness. The authors also took a cognitive neuroscience perspective and evaluated how differences may occur in the way that food-intrinsic and extrinsic information become integrated with sweetness perception [1]. Based on recent neuroscientific evidence, the authors proposed a new framework of multisensory flavor integration focusing not on the food-intrinsic/extrinsic divide, but rather on whether the sensory information is perceived to originate from within or outside the body. In conclusion, Wang et al. (2020) provided recommendations to those in the food industry and proposed directions for future research relating to the need for longer-term studies and understanding of individual differences.

## 3. Conclusions

Overall, the works included in this Special Issue collection are diverse, and cover a wide range of studies from fundamental to real world applicability re consumer preference and acceptance. A theming of the studies has been utilized to emphasize the diverse and critical nature of the inclusion of the human senses in consumer and acceptance applications across the food stakeholder chain. Of note is that many of the studies utilize unique multidisciplinary study design approaches and methodologies and involve synergy in disciplines. An overall conclusion with respect to this anthology is that the human senses, consumer acceptance and preferences are core to future food design regarding understanding numerous fundamental and applicable settings involving human perception in the food space.
